# The Chemistry of Sustainable Energy Conversion and Storage

**DOI:** 10.3390/molecules27123731

**Published:** 2022-06-10

**Authors:** Guanglin Xia

**Affiliations:** Department of Materials Science, Fudan University, Shanghai 200433, China; xiaguanglin@fudan.edu.cn

The long-term environmental side-effects and finite supply of fossil fuels, which dominate the energy resources in our daily lives, require a transition to renewable and clean energy resources. Renewable energy sources, such as solar, wind, and hydro, hold great promise to meet the huge energy demands of the 21st century at no environmental cost. Utilizing these energies, however, requires efficient and low-cost energy conversion and storage techniques, whose performance directly relies on the related chemistry during the conversion and storage process. Excitingly, owing to the advancement of materials synthesis, chemical modifications, and characterization techniques, the chemistry behind sustainable energy conversion and storage has been greatly improved, and hence, the performance of various energy conversion and storage devices has been effectively enhanced.

Herein, this Special Issue, including eight research articles and one review, provides a better understanding of the related chemistry behind various energy conversion and storage techniques. Saruhan et al. reported the fabrication of micro-supercapacitors using laser-induced interdigital structured graphene electrodes, which delivered an energy density of 0.256 μWh cm^−2^ at 0.03 mA cm^−2^ and power density of 0.11 mW cm^−2^ at 0.1 mA cm^−2^ [1]. More importantly, excellent cycling stability up to 10,000 cycles could be realized, which demonstrates its potential in terms of peak energy storage ability. Pant et al. adopted a personal computer one-dimensional simulation to validate the possible application of ultrathin layering and the high efficiency of graphene as a back surface field based on a CdTe solar cell [2]. Kustov et al. investigated the adoption of alumina-supported Co and Fe catalysts in the reduction of CO_2_ with hydrogen into CO under supercritical conditions [3]. It was demonstrated that the differences in the crystallographic phase features of Fe-containing catalysts cause the reverse water gas shift reaction to form carbon monoxide and the reduced iron phases would produce a mixture of hydrocarbons via the Fischer–Tropsch reaction. Wang et al. discovered a Cu_2_O-Ag tandem catalyst for a synergetic electrochemical carbon dioxide reduction reaction to C_2_ chemicals [4]. It is theoretically and experimentally demonstrated that the introduction of Ag enhances the CO concentration on the surface of Cu_2_O and meanwhile reduces the C-C coupling reaction barrier energy, which leads to the improved synthesis of C_2_ products. In addition, two bifunctional catalysts, i.e., Ni/WO_3_-TiO_2_ and Ni/WO_3_-ZrO_2_, that exhibit excellent performance in glycerol conversion into C_3_ alcohols were reported [5]. The total yield of 1-propanol and 2-propanol reaches 95%, and the glycerol conversion is even close to 100% at 250 °C under a hydrogen pressure of 3 MPa. Kuo et al. reported the successful synthesis of two types of meso/microporous carbon materials based on the carbonization and the activation of two different kinds of hyper-crosslinked polymers [6]. They exhibited a high specific area of up to 1100 m^2^ g^−1^ and hence displayed excellent CO_2_ uptake performance. Moreover, a high specific capacitance of 453 F g^−1^ at 5 mV s^−1^ could be achieved when using these carbon materials in supercapacitors. Serrano-Ruiz et al. realized the improved thermochemical energy storage behavior of Mn_2_O_3_ via the introduction of MoO_3_ based on a precipitation method [7]. Mn_2_O_3_ composed of 0.6 and 1.5 wt.% of MoO_3_ exhibited outstanding cycling stability after 45 consecutive reduction-oxidation cycles at a high temperature of 600~1000 °C. To decrease the electron transfer depletion in/on the pseudocapacitive electrode, Zheng et al. fabricated a novel structure composed of Co nanoparticles embedded in MnO nanowires with an N-doped carbon coating on carbon cloth [8]. This electrode exhibits a high specific capacitance of 747 F g^−1^ at 1 A g^−1^ and good cycling stability with 93% capacitance retention after 5000 cycles at 10 A g^−1^. Furthermore, considering the unique properties of bismuth chalcogenide as anode materials of Li-ion batteries, Jain et al. summarized the main issues and recent advances associated with bismuth chalcogenide anodes using both solid and liquid electrolytes [9].

In summary, the development of sustainable energy conversion and storage devices has been a hot research topic across the world, and the chemical reaction behind these devices plays an important role in further improving their performance. All the articles presented in this Special Issue contribute to enhancing our understanding of the chemical mechanism of various energy conversion and storage devices. As a result, I would like to thank all the authors for their valuable contributions to this Special Issue and all the peer reviewers for their valuable comments. Last but not least, I would like to thank all the staff members of MDPI for their editorial support in preparing this Special Issue.

## Data Availability

Not applicable.

## References

[1] Ray A., Roth J., Saruhan B. (2022). Laser-Induced Interdigital Structured Graphene Electrodes Based Flexible Micro-Supercapacitor for Efficient Peak Energy Storage. Molecules.

[2] Devendra K.C., Shah D.K., Akhtar M.S., Park M., Kim C.Y., Yang O.B., Pant B. (2021). Numerical Investigation of Graphene as a Back Surface Field Layer on the Performance of Cadmium Telluride Solar Cell. Molecules.

[3] Bogdan V.I., Koklin A.E., Kustov A.L., Pokusaeva Y.A., Bogdan T.V., Kustov L.M. (2021). Carbon Dioxide Reduction with Hydrogen on Fe, Co Supported Alumina and Carbon Catalysts under Supercritical Conditions. Molecules.

[4] Niu D., Wei C., Lu Z., Fang Y.Y., Liu B., Sun D., Hao X.B., Pan H.G., Wang G.M. (2021). Cu_2_O-Ag Tandem Catalysts for Selective Electrochemical Reduction of CO_2_ to C_2_ Products. Molecules.

[5] Greish A.A., Finashina E.D., Takachenko O.P., Kustov L.M. (2021). Preparation of Propanols by Glycerol Hydrogenolysis over Bifunctional Nickel-Containing Catalysts. Molecules.

[6] Mohamed M.G., Ahmed M.M.M., Du W.T., Kuo S.W. (2021). Meso/Microporous Carbons from Conjugated Hyper-Crosslinked Polymers Based on Tetraphenylethene for High-Performance CO_2_ Capture and Supercapacitor. Molecules.

[7] Moya J., Marugán J., Orfila M., Díaz-Pérez M.A., Serrano-Ruiz J.C. (2021). Improved Thermochemical Energy Storage Behavior of Manganese Oxide by Molybdenum Doping. Molecules.

[8] Chen G.Q., Zhang X., Ma Y., Song H., Pi C., Zheng Y., Gao B., Fu J., Chu P.K. (2020). In-Situ Synthesis of Heterostructured Carbon-Coated Co/MnO Nanowire Arrays for High-Performance Anodes in Asymmetric Supercapacitors. Molecules.

[9] Singh R., Kumari P., Kumar M., Ichikawa T., Jain A. (2020). Implementation of Bismuth Chalcogenides as an Efficient Anode: A Journey from Conventional Liquid Electrolyte to an All-Solid-State Li-Ion Battery. Molecules.

